# Optimizing Sequential and Combined Anabolic and Antiresorptive Osteoporosis Therapy

**DOI:** 10.1002/jbm4.10041

**Published:** 2018-02-27

**Authors:** Benjamin Z Leder

**Affiliations:** ^1^ Harvard Medical School Boston MA USA; ^2^ Endocrine Unit Massachusetts General Hospital Boston MA USA

**Keywords:** ANABOLICS, THERAPEUTICS, ANTIRESORPTIVES, DXA, ANALYSIS/QUANTITATION OF BONE, BIOCHEMICAL MARKERS OF BONE TURNOVER, BONE MODELING AND REMODELING, OSTEOPOROSIS, DISEASES AND DISORDERS OF/RELATED TO BONE

## Abstract

As osteoporosis therapy options have expanded, and clinical guidelines have begun to embrace the concept of limited treatment courses and “drug holidays,” the choices that physicians must make when initiating, electing to continue, or switching therapies have become more complex. As a result, one of the fundamental issues that must be carefully considered is whether, when, and in what sequence anabolic therapies should be utilized. This review evaluates the current evidence supporting the optimal sequence for the use of anabolic and antiresorptive drugs and assesses the expanding number of clinical trials favoring the initial use of anabolic therapy followed by an antiresorptive agent. This review also explores the evidence suggesting that the effectiveness of anabolic medications are diminished when used in patients that have been previously treated with specific antiresorptive drugs for prolonged periods. Finally, the recent advances in designing combination antiresorptive/anabolic treatment approaches are detailed, with a focus on combined denosumab/teriparatide regimens, which appear to provide the most substantial and clinically relevant skeletal benefits to patients with established osteoporosis. © 2018 The Authors. JBMR Plus is published by Wiley Periodicals, Inc. on behalf of the American Society for Bone and Mineral Research.

## Introduction

Although osteoporosis therapy has advanced substantially over the past two decades, our currently available antiresorptive and anabolic medications can at best increase bone mineral density (BMD) modestly and reduce nonvertebral fracture rates by 20% to 40%.^1^, ^2^, ^3^, ^4^, ^5^ Thus, the need for more effective therapeutic regimens remains pressing, especially for those at the highest risk of fragility fracture. An additional challenge in managing patients with established osteoporosis is the increasing reluctance to treat patients with antiresorptive medications for more than 3 to 5 years because of the concern over uncommon but serious side effects such as atypical femur fracture and osteonecrosis of the jaw, as well as the long‐standing regulatory 2‐year limit on parathyroid‐hormone receptor targeted anabolic therapies.^6^, ^7^, ^8^ Thus, it is expected that over a lifetime, the use of more than one medication will be required for many patients with established disease. And consequently, it is imperative that we understand the selective effects of osteoporosis medications when used sequentially or in combination so that we can construct optimal treatment plans in individual patients. This review evaluates the available evidence concerning the differential clinical effects of the various sequential and combination osteoporosis treatment approaches that have been investigated to date and details some of the important pharmacological distinctions between these approaches.

### Current Osteoporosis Drugs: Comparative Mechanisms

Although a detailed discussion of the mechanisms of action of osteoporosis therapies is beyond the scope of this review, the differences between these mechanisms impacts the differences observed with the various sequential and combination approaches.

### Anabolic therapies

Currently, the two approved anabolic osteoporosis medications (teriparatide, a parathyroid hormone [PTH] analog, and abaloparatide, a parathyroid hormone–related peptide [PTHrP] analog) both exert their effects through binding to the PTH/PTHrP receptor, which is expressed in many tissues including osteoblasts, osteocytes, and renal tubule cells.^9^ The anabolic pharmacologic efficacy of PTH and PTHrP analogs appears to be dependent on intermittent administration as sustained receptor stimulation enhances the effects on bone resorption.^10^ The molecular mechanisms that underlie the net anabolic activity of PTH and PTHrP analogs when given intermittently, however, are still being defined. Specifically, one of the principal questions that remains unanswered is what portion of the anabolic effects of these drugs are mediated through the initial stimulation of bone resorption (through the release of growth factors from the skeletal matrix) as opposed to direct effects on osteoblasts (decreased apoptosis), osteocytes (decreased sclerostin production), and lining cells (restored anabolic activity).^11^ Whatever the relative importance of these mechanisms, however, the net effect of PTH and PTHrP analogs on the human skeleton is to increase trabecular bone mass and improve trabecular microarchitecture while concomitantly increasing cortical bone porosity.^12^, ^13^, ^14^, ^15^ Despite the increase in porosity, the cumulative effects of these drugs are to increase bone strength and decrease fracture incidence.^4^, ^5^, ^16^, ^17^, ^18^, ^19^, ^20^, ^21^ More recently, it has also been suggested that the relative pharmacological effects of PTH/PTHrP analogs may differ based on their relative binding affinities to different PTH/PTHrP receptor conformations. Specifically, in vitro and animal studies suggest that PTH/PTHrP receptor ligands can distinguish between two distinct receptor conformations—labeled R^0^ and RG—and that more efficient binding to R^0^ results in prolonged signaling and a greater calcemic response whereas more efficient binding to RG results in a more transient response.^22^, ^23^ It is thus possible, though at this point unproven, that the observed differential binding affinities of abaloparatide and teriparatide to the RG conformation of the PTH/PTHrP receptor may account for some of the observed differences in bone resorption rates and hypercalcemia incidence between these two agents.^24^

Romosozumab is an investigational monoclonal antibody that inhibits sclerostin, an osteocyte‐secreted protein that acts as an extracellular inhibitor of canonical Wnt signaling by binding to lipoprotein receptor‐related proteins LRP4, LRP5, and LRP6.^25^ Sclerostin inhibits the proliferation, differentiation, and survival of osteoblasts (hence inhibiting bone formation) and upregulates osteocytic receptor activator of nuclear factor‐κB (RANK) ligand (RANKL) synthesis (thereby stimulating bone resorption).^26^, ^27^ By inhibiting sclerostin activity romosozumab transiently stimulates bone formation while decreasing bone resorption in a more sustained fashion, resulting in improved skeletal integrity and fracture reduction.^28^, ^29^, ^30^, ^31^ Romosozumab has also been shown to reduce fracture incidence more than the most commonly used bisphosphonate (alendronate), though its current potential as an osteoporosis therapy remains unclear because of cardiovascular safety concerns in humans.^32^

### Antiresorptive therapies

The most commonly used antiresorptive medications are nitrogen‐containing bisphosphonates. These drugs act by binding to hydroxyapatite and inhibiting the enzyme farnesyl diphosphate synthase in the cholesterol biosynthetic pathway, suppressing protein geranylgeranylation, and hence osteoclastic bone resorption (as well as coupled bone formation).^33^ Despite common mechanisms of action, however, the different bisphosphonates (which include the commonly prescribed alendronate, risedronate, ibandronate, and zoledronic acid) differ significantly in terms of both anti‐remodeling potency and the degree of persistence in the skeletal matrix.^34^ Denosumab is a monoclonal antibody that blocks the binding of RANKL to its osteoclast‐derived receptor, RANK, thus inhibiting osteoclast formation, activation, and survival and hence bone resorption.^3^, ^35^ Denosumab is the most rapidly acting and potent antiresorptive agent currently in use, but its effects are rapidly reversible and when discontinued, bone turnover rates increase to levels above the pretreatment baseline.^36^, ^37^, ^38^, ^39^, ^40^, ^41^ Importantly, this post‐denosumab “rebound” phenomenon has been linked to an increased incidence of multiple vertebral fractures and may also contribute to the observed maladaptive changes in bone turnover and bone loss that occurs in patients transitioning from denosumab to PTH/PTHrP‐analog therapy (discussed in the “Anabolic Agents After Antiresorptive Agents” section below).^42^, ^43^, ^44^, ^45^, ^46^, ^47^, ^48^, ^49^ Estrogens and selective estrogen receptor modulators, primarily acting through its binding to estrogen receptor alpha (ERα), play a key role in both osteoblast and osteoclast biology but in the pharmacologic setting act primarily as antiresorptive agents.^50^ The antiresorptive effects of these drugs are thought to rely on several molecular mechanisms including suppressing stromal cell, osteoblast, and lining cell production of RANKL, increasing osteoblastic production of osteoprotegerin, directly suppressing the production of proresorptive cytokines, and promoting osteoclast apoptosis.^50^, ^51^ Like denosumab, the bone turnover suppressive effects of these compounds are rapidly reversible, though a rebound phenomenon is not prominent.^52^, ^53^, ^54^

### Antiresorptive Agents After Anabolic Agents

When teriparatide therapy is initiated and sustained, serum and urine markers of bone remodeling generally return to their pretreatment baseline before the end of the 24‐month course while BMD continues to increase over the entire treatment period. This apparent discrepancy may be explained by the ability of teriparatide to continue to stimulate modeling‐based bone formation even as remodeling rates revert to baseline (as has been suggested in a recent histomorphometric analysis).^55^ When teriparatide is discontinued, however, BMD quickly decreases (though faster in postmenopausal women compared to eugonadal men).^56^ And although studies have suggested that some antifracture efficacy may be maintained for up to 18 months after the drug has been stopped,^57^ it is likely that most of the beneficial effects do eventually dissipate.

**Figure 1:**
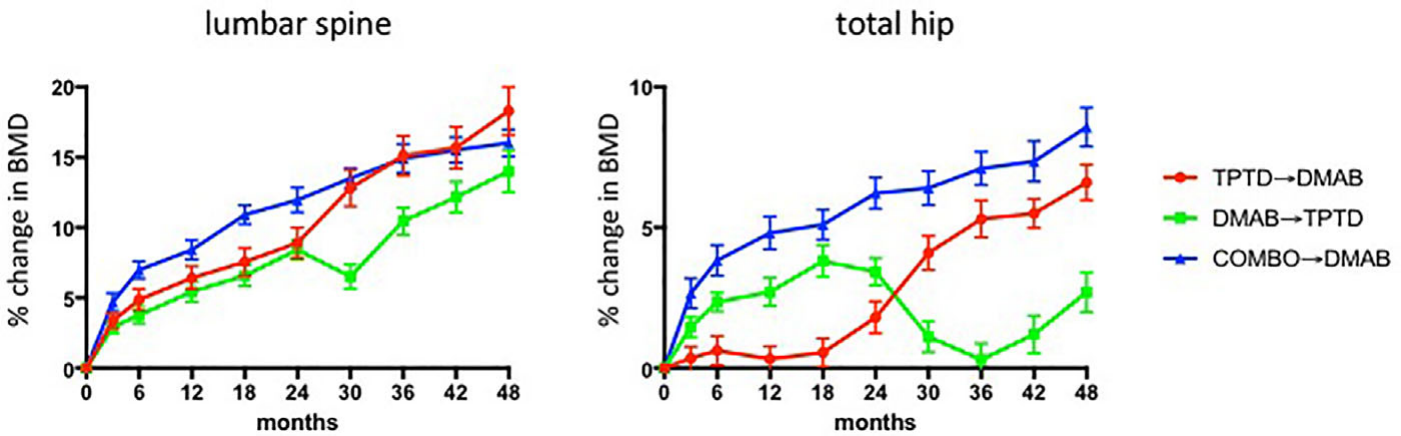
Change in lumbar sine and total hip BMD in osteoporotic women who received 2 years of teriparatide followed by 2 years of denosumab (red circles), 2 years of denosumab followed by 2 years of teriparatide (green squares), and 2 years of both drugs followed by 2 years of denosumab (blue triangles). All groups differ significantly at the hip, but not the spine, at month 48. (Adapted from Leder and colleagues.^43^)

Numerous studies have investigated strategies to maintain teriparatide‐induced gains in bone mass after the drug is discontinued. Oral alendronate was studied in several clinical trials and is clearly effective not only in preventing post‐teriparatide and post‐PTH bone loss but also in further increasing hip and spine BMD.^58^, ^59^, ^60^ The selective estrogen receptor modulator, raloxifene, also appears to prevent post‐teriparatide bone loss but may be somewhat less effective at increasing BMD, particularly at the spine.^61^ More recently, in the DATA‐Switch study, 2 years of denosumab when given after 2 years of teriparatide increased spine BMD by an additional 9.4% (18.3% total 4‐year increase) and increased total hip BMD an additional 4.8% (6.6% total 4‐year increase), gains that appear to be significantly greater than what can be achieved with bisphosphonates therapy after teriparatide (Fig. 1).^43^, ^62^ Moreover, denosumab was also able to further increase BMD in patients who previously received 2 years of combined teriparatide/denosumab therapy.^43^ Similarly, patients who have been treated with 18 months of abaloparatide experience further BMD gains and maintain a fracture‐reduction benefit when switched to 6 months of alendronate (extended to 24 months in an unpublished abstract).^63^ Finally, the effects of antiresorptive therapy after romosozumab has also been studied in large randomized trails with both alendronate and denosumab demonstrating the capacity to both increase BMD and maintain antifracture efficacy.^28^, ^32^ Taken together, these studies strongly suggest that antiresorptive drugs routinely be prescribed when a course of anabolic therapy is concluded.

### Anabolic Agents After Antiresorptive Agents

Although randomized controlled trials of anabolic agents have largely excluded patients who have received recent antiresorptive therapy, in clinical care it is often the case that patients who are prescribed these drugs have had extensive and often prolonged exposure to antiresorptives, including bisphosphonates. Given that bisphosphonates are imbedded in the bone matrix and are able to recirculate in the matrix for many years,^34^ it is has long been hypothesized that prior bisphosphonate use could influence the subsequent response to anabolic agents, particularly PTH analogs whose mechanism of action is partially dependent on stimulating bone resorption. Indeed, most studies have confirmed that there is some blunting in BMD increases when teriparatide is given to patients who were previously treated with bisphosphonates (more at the hip than the spine).^61^, ^64^, ^65^, ^66^ Moreover, studies have suggested that bisphosphonates with longer skeletal half‐lives may produce a more pronounced blunting than those with more transient biological activity.^66^, ^67^ More recently, the post‐bisphosphonate skeletal effects of romosozumab were directly compared to teriparatide in a randomized open label trial of postmenopausal osteoporotic women (all subjects had taken bisphosphonates for at least 3 years and alendronate during the year prior to enrollment).^68^ In women randomized to teriparatide, hip BMD decreased slightly over 12 months whereas it increased by approximately 3% in those receiving romosozumab. Spine BMD increased in both groups, but significantly more in those treated with romosozumab. Notably, however, the romosozumab‐induced gains in hip and spine BMD appear to be substantially smaller than those observed in separate studies of treatment‐naive subjects or subjects with only remote bisphosphonate use.^28^, ^29^, ^32^

The transition from denosumab‐to‐teriparatide follows a pattern that differs from the transition from bisphosphonates to teriparatide. In the DATA‐Switch study described in the previous section, women who received 2 years of denosumab and were then switched to 2 years of teriparatide experienced a 6‐month decline in spine BMD but more extensive and progressive bone loss at the hip and distal radius (Fig. 1).^43^ Moreover, switching from denosumab to teriparatide resulted in extremely accelerated bone remodeling as evidenced by sustained increases in serum osteocalcin and C‐telopeptide to levels greater than 200% above their original baseline.^43^ The mechanism by which teriparatide exerts this extensive pro‐remodeling effect in patients is undefined but may relate to teriparatide stimulating a large pool of previously quiescent osteoclast precursors in a synchronized fashion. Given the recent suggestion that the accelerated bone remodeling which occurs when denosumab is discontinued (even in the absence of adding a pro‐remodeling drug) is associated with an increase risk of compound vertebral fractures, it seems prudent to suggest that physicians avoid prescribing this specific drug transition.^49^, ^69^

Indeed, it has been suggested that the discontinuation of denosumab should be uniformly followed by a bisphosphonate (despite only limited evidence of the utility of this approach at present).^70^, ^71^, ^72^ Unfortunately, there are currently no trials addressing the optimal timing of bisphosphonate therapy initiation after denosumab or which bisphosphonate to choose. Given bisphosphonates’ mechanism of action and preferential deposition at sites of active bone remodeling,^73^ however, it is plausible that if the bisphosphonate is administered while denosumab's antiresorptive effects are still maximal, the drug may be less effective than if given to a patient in whom the effects of denosumab are no longer evident and robust bone turnover is ongoing. This issue may be irrelevant if an oral bisphosphonate is being repeatedly administered weekly or monthly, but may be quite relevant to patients transitioning from denosumab to yearly zoledronic acid.

### Antiresorptive and Anabolic Agents in Combination

Unlike most common medical conditions, such as hypertension or diabetes, there is not yet a universally accepted role for using more than one drug at a time in the treatment of osteoporosis. Early studies investigating the effects of combining two antiresorptive agents did not show a significant benefit.^74^ More recently, a series of clinical trials combining PTH analogs with antiresorptive medications have been performed, some with encouraging results (combination studies using abaloparatide or romosozumab have not been reported). The first clinical studies involving combined antiresorptive/anabolic therapy involved the co‐administration of estrogen or selective estrogen receptor modulators and teriparatide but either lacked monotherapy comparison groups or were of short duration and thus are difficult to interpret.^75^, ^76^, ^77^, ^78^, ^79^, ^80^, ^81^, ^82^

**Figure 2:**
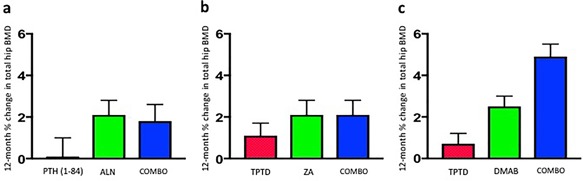
One‐year change in total hip BMD in osteoporotic women treated with (*A*) PTH (1‐84), alendronate (ALN) and both drugs (COMBO), (*B*) teriparatide (TPTD), zoledronic acid (ZA) and both drugs (COMBO), and (*C*) teriparatide (TPTD), denosumab (DMAB) and both drugs (COMBO). (Adapted from Reeve and colleagues,^75^ Cosman and colleagues,^79^ and Finkelstein and colleagues.^90^)

Alendronate was the first bisphosphonate studied in combination with PTH analogs in humans. In the PATH study, postmenopausal osteoporotic women were randomized to receive PTH (1‐84), alendronate, or the combination for 12 months. Lumbar spine BMD increased similarly in the combination, PTH monotherapy, and alendronate groups, whereas hip BMD increased more in women receiving both medications than those receiving PTH alone but less (though not significantly less) than those receiving alendronate alone (Fig. 2).^83^ Moreover, QCT‐derived volumetric trabecular BMD of the spine increased by double the percentage in the PTH group compared to the combined group, a clear demonstration of blunting in the trabecular compartment. In a separate study, postmenopausal women with osteoporosis were randomized to receive either alendronate, teriparatide (at double the FDA‐approved dose), or both for 30 months (teriparatide was not started until month 6) and hip and spine BMD increased more in women treated with teriparatide alone than with combination therapy.^84^ In a 12‐month randomized controlled trial, postmenopausal women with osteoporosis were randomized to a single infusion of zoledronic acid 5 mg, teriparatide, or the combination of both medications, and lumbar spine BMD increased similarly in the combination group and the teriparatide monotherapy group (and more than the zoledronic acid monotherapy group) while total hip and femoral neck BMD increased similarly in the combination group and the zoledronic acid monotherapy group (and more than the teriparatide monotherapy group) (Fig. 2).^85^ Additional studies have investigated alternate combination treatment strategies, including those in which alendronate was added to ongoing teriparatide^86^, ^87^ or teriparatide was added to ongoing alendronate.^88^ In the former study, spine and hip BMD increased more when alendronate was added than when teriparatide monotherapy was continued (though no comparison was made to switching from teriparatide to alendronate); and in the latter study, spine and hip BMD increased more when teriparatide was added to alendronate than when alendronate was discontinued before teriparatide was started (though no comparison was made to simply continuing alendronate). When these bisphosphonate/PTH analog studies are taken together with additional combination therapy studies, including those performed in men, the data suggests that the co‐administration of bisphosphonates and PTH analogs does not provide substantial clinical benefits compared to monotherapy, although approaches of adding one class of agent to patients being treated with the other deserve further study.^89^, ^90^, ^91^, ^92^

**Figure 3:**
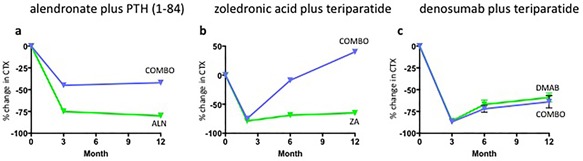
One‐year change in CTX in osteoporotic women treated with (*A*) combined PTH (1‐84) and alendronate (ALN), (*B*) combined teriparatide and zoledronic acid (ZA), and (*C*) combined teriparatide and denosumab (DMAB). (Adapted from Reeve and colleagues,^75^ Cosman and colleagues,^79^ and Finkelstein and colleagues.^90^)

The most promising combination approach tested to date is the concomitant use of teriparatide and denosumab. This conclusion is based on the results of the DATA study in which 94 postmenopausal women with osteoporosis were randomized to receive teriparatide, denosumab, or both medications for 24 months.^93^, ^94^ Unlike the combination of bisphosphonates and teriparatide, spine and hip BMD increased significantly more in those treated with both drugs compared to either drug alone. Moreover, most of the benefit of combination therapy was apparent during the first 12 months of treatment, during which spine BMD increased by over 9% in the combination group versus approximately 6% in the teriparatide or denosumab groups, and total hip BMD increased by approximately 5% in the combination group compared to <1% and 2% in the teriparatide and denosumab groups, respectively (Fig. 2). Additionally, radius and tibia HR‐pQCT‐assessed cortical volumetric BMD, cortical thickness, and estimated bone strength increased more in women treated with combined denosumab plus teriparatide than either monotherapy group while cortical porosity, which progressively increased in women treated with teriparatide alone over the full 24 months, remained stable in women treated with both drugs.^(20)^ Interestingly, bone resorption markers were identical in patients treated with denosumab alone and those treated with combination therapy, whereas markers of bone formation were more suppressed in those treated with denosumab alone than those receiving both drugs, especially at the early time points. These patterns differ significantly from those observed with bisphosphonate‐containing combinations in which teriparatide is still able to stimulate bone resorption even in the presence of the antiresorptive drug (Fig. 3). Together, these findings suggest that the unique efficacy of combined denosumab and teriparatide may be related to denosumab's ability to fully block the proresorptive effects of teriparatide while still allowing for teriparatide‐induced stimulation of modeling‐based bone formation.

## Summary

As osteoporosis treatment options have expanded, the choice of which medication to prescribe and in what order has become more complicated. Nonetheless, recent studies support the emergence of several themes that can help guide our decision‐making. Among these themes are the fundamental observations that in patients for whom more than one drug will be required over an extended period, the greatest gains in bone mass can be achieved with the initial use of an anabolic agent followed by an antiresorptive drug whereas the initial use of a bisphosphonate may diminish the efficacy of subsequent anabolic therapy. Moreover, it has also become evident that the specific transition from denosumab to PTH analogs should be avoided because of the resultant accelerated bone turnover and sustained bone loss. Finally, although there is still no fracture data to support the use of combination anabolic/antiresorptive therapy, the combination of denosumab and teriparatide shows promise and may be considered in patients who are at the highest risk of fragility fracture.

## Disclosures

Research funding from Amgen and Lilly. Consultant Amgen, Radius.
